# Understanding the Impact of Hydrogen Activation by SrCe_0.8_Zr_0.2_O_3−δ_ Perovskite Membrane Material on Direct Non-Oxidative Methane Conversion

**DOI:** 10.3389/fchem.2021.806464

**Published:** 2022-01-10

**Authors:** Sichao Cheng, Su Cheun Oh, Mann Sakbodin, Limei Qiu, Yuxia Diao, Dongxia Liu

**Affiliations:** ^1^ Department of Chemical and Biomolecular Engineering, University of Maryland, College Park, MD, United States; ^2^ Research Institute of Petroleum Processing, SINOPEC, Beijing, China

**Keywords:** iron/silica catalyst, perovskite membrane, direct non-oxidative methane conversion, coke formation, mixed ionic-electronic conductor

## Abstract

Direct non-oxidative methane conversion (DNMC) converts methane (CH_4_) in one step to olefin and aromatic hydrocarbons and hydrogen (H_2_) co-product. Membrane reactors comprising methane activation catalysts and H_2_-permeable membranes can enhance methane conversion by *in situ* H_2_ removal via Le Chatelier's principle. Rigorous description of H_2_ kinetic effects on both membrane and catalyst materials in the membrane reactor, however, has been rarely studied. In this work, we report the impact of hydrogen activation by hydrogen-permeable SrCe_0.8_Zr_0.2_O_3−δ_ (SCZO) perovskite oxide material on DNMC over an iron/silica catalyst. The SCZO oxide has mixed ionic and electronic conductivity and is capable of H_2_ activation into protons and electrons for H_2_ permeation. In the fixed-bed reactor packed with a mixture of SCZO oxide and iron/silica catalyst, stable and high methane conversion and low coke selectivity in DNMC was achieved by co-feeding of H_2_ in methane stream. The characterizations show that SCZO activates H_2_ to favor “soft coke” formation on the catalyst. The SCZO could absorb H_2_ *in situ* to lower its local concentration to mitigate the reverse reaction of DNMC in the tested conditions. The co-existence of H_2_ co-feed, SCZO oxide, and DNMC catalyst in the present study mimics the conditions of DNMC in the H_2_-permeable SCZO membrane reactor. The findings in this work offer the mechanistic understanding of and guidance for the design of H_2_-permeable membrane reactors for DNMC and other alkane dehydrogenation reactions.

## Introduction

Direct non-oxidative methane conversion (DNMC) has received intense attention in the past decades since it directly converts methane into value-added hydrocarbons such as ethylene (C_2_H_4_) and benzene (C_6_H_6_), and hydrogen (H_2_) co-product (Borry et al., 1999; Lunsford, 2000; Xu et al., 2003; Alvarez-Galvan et al., 2011). However, there are inherent challenges in the DNMC reaction such as low thermodynamic equilibrium conversion and catalyst deactivation due to the reaction endothermicity and coke deposition, respectively (Spivey and Hutchings, 2014). A strategy to increase methane conversion in DNMC is to conduct the reaction in a hydrogen-permeable membrane reactor (Oh et al., 2019; Liu et al., 2020). Hydrogen (H_2_) is the smallest molecule in DNMC, and its yield reaches up to ∼50% in the product effluent, which directly influences the kinetics and thermodynamics of the reaction. According to the Le Chatelier's principle, the removal of hydrogen produced in DNMC can shift the thermodynamic equilibrium to higher methane conversion. Membrane reactors comprising methane activation catalysts and H_2_-permeable membranes, therefore, have been intensively studied since the 1990s (Li et al., 2001; Li et al., 2002; Morejudo et al., 2016)

Given the reaction temperature (typically, >873 K) of DNMC, research has been focusing on thermally and chemically stable metal and ceramic membrane materials integrated with molybdenum/zeolite (e.g., Mo/ZSM-5) (Wang et al., 1993) or iron/silica (Fe/SiO_2_) (Guo et al., 2014) catalysts in membrane reactor studies. For example, Larachi's group had developed a palladium-silver (Pd-Ag) alloy membrane on porous stainless steel support for direct DNMC over a zeolite supported ruthenium-molybdenum (i.e., Ru-Mo/ZSM-5) catalyst at temperatures up to 973 K (Iliuta et al., 2003). The catalytic performance tests showed that the Pd-alloy membrane was effective in hydrogen permeation and resulted in a significant increase in methane conversion. Similarly, Morreale and co-workers had fabricated the Pd membranes containing a packed Mo/ZSM-5 catalyst for DNMC (Natesakhawat et al., 2015) to achieve significant improvement in methane conversion and total aromatics yield through *in situ* H_2_ removal. For ceramic membrane reactors for DNMC, perovskites-based metal oxides that have mixed ionic-electronic conductivity (MIEC) were used as membrane materials in DNMC. For instance, Iglesia's group had manufactured supported SrCe_0.95_Yb_0.05_O_3-δ_ thin membrane for DNMC over the Mo/ZSM-5 catalyst. The reactor demonstrated a slight increase in methane conversion due to H_2_ removal (Liu et al., 2002b; Hamakawa et al., 2002). It should be noted that all these studies were based on the metal/zeolite catalyst systems which yielded a higher amount of coke and accelerated catalyst deactivation in the membrane reactor tests.

Our group has recently developed a H_2_-permeable SrCe_0.8_Zr_0.2_O_3−δ_ (SCZO) perovskite-based membrane reactor for DNMC over the Fe/SiO_2_ catalyst, which exhibited significantly higher stability and activity than the membrane reactors packed with the traditional Mo/ZSM-5 catalyst (Sakbodin et al., 2016). The SCZO-based membrane reactor also achieved high methane conversion and long-term stability and was chemically and thermally stable at high-temperature conditions of Fe/SiO_2_ catalyst functioning when DNMC was coupled with either hydrogen combustion or reverse water gas shift reaction (Sakbodin et al., 2020; Sakbodin et al., 2021a; Sakbodin et al., 2021b). As expected, the products were shifted to heavier hydrocarbons such as naphthalene when the DNMC was run in the SCZO-based membrane reactor for hydrogen removal. Moreover, we studied the addition of H_2_ into the reaction zone via the SCZO membrane by flowing H_2_ sweep in the membrane reactor (Sakbodin et al., 2016). It shows higher methane conversion than the fixed-bed reactor setting; besides, the product selectivity was modulated to lighter hydrocarbons. Clearly, we achieved the tuning of the product selectivity towards lighter hydrocarbons without sacrificing the methane conversion, which was distinct from all previous H_2_ co-feed studies in the fixed-bed reactor conditions in literature.

Inspired by the tailorability of product selectivity without sacrifice of methane conversion in DNMC in membrane reactors by H_2_ addition into the reaction zone via H_2_-permeable SCZO perovskite, we aimed to provide mechanistic understanding of the impacts of hydrogen addition on DNMC in the presence of the H_2_-permeable membrane and methane activation catalyst materials. Hydrogen is the co-product of dehydrogenation of alkane, whose presence impacts both the reaction kinetics and thermodynamics of DNMC. Studies for DNMC in the fix-bed reactors have shown that addition of a significant amount of hydrogen co-feed would reduce methane conversion, while a small amount would have favorable effect in terms of catalyst stability and lighter hydrocarbon production (Liu et al., 2002c; Ma et al., 2003; Osawa et al., 2003; Ma et al., 2005; Kojima et al., 2006; Aritani et al., 2009). For example, it was proposed that 3–6% of H_2_ suppressed coke deposition on a 6 wt% Mo/HZSM-5 catalyst in a DNMC reaction (Ma et al., 2003; Ma et al., 2005; Kojima et al., 2006). In the MIEC ceramic membranes, hydrogen is permeated electrochemically (Kreuer, 2003; Phair and Badwal, 2006; Fabbri et al., 2010). First, hydrogen is dissociated and ionized to form the hydroxide defects (proton defects), which migrates through the membrane via proton hopping between adjacent oxygen ions at normal lattice sites. At the permeate side of the membrane, the defects are then reduced to form hydrogen molecules and desorbed from the membrane surface. The formation of hydrogen species upon activation in the SCZO material is expected to influence the DNMC in different manner than that of H_2_ co-feed in DNMC without the MIEC materials.

Herein, we report the performance of DNMC over the SCZO perovskite oxide, Fe/SiO_2_ catalyst, and a mixture of both SCZO oxide and Fe/SiO_2_ catalyst in the absence and presence of H_2_ co-feed in methane stream, respectively. The methane conversion, product selectivity, and coke formation in all these cases were evaluated and compared. In addition, the properties of coke formed on SCZO membrane, Fe/SiO_2_ catalyst, and their mixture were analyzed using Raman spectroscopy, X-ray photoelectron spectroscopy (XPS), and temperature programmed oxidation (TPO) techniques. The present study rigorously analyzed the impact of H_2_ activation by H_2_-permeable SCZO perovskite oxide on methane activation catalysts in DNMC, a topic that has been rarely explored in H_2_-permeable membrane reactor literature in the past years.

## Experimental

### Synthesis of Membrane and Catalyst Materials

The SCZO perovskite oxide material was prepared by a conventional solid-state synthesis method (Sakbodin et al., 2016). In the synthesis process, stoichiometric amounts of strontium carbonate (SrCO_3_, ≥99.9% purity, Sigma-Aldrich), cerium oxide (CeO_2_, 99.9% purity, Alfa Aesar), and zirconium oxide (ZrO_2_, 99.9% purity, Inframat) were ball-milled to ensure even mixing of the starting chemicals. In order to obtain homogeneous solution in the ball milling process, ethanol (200 proof, Pharmco) and milling media (yttria-stabilized zirconia) were adequately added. The resultant slurry was ball milled for 24 h, followed by drying and grinding into fine powder and then calcination at 1573 K for 10 h. The as-obtained material was SCZO perovskite ceramic powder and was directly used in the catalysis tests.

The Fe/SiO_2_ catalyst material was prepared by fusing iron silicate (Fe_2_SiO_4_) and quartz particles (SiO_2_, BDH) at 1973 K for 6 h in stagnant air in a high-temperature furnace (MTI Corporation KSL1700X), as reported in our previous work (Sakbodin et al., 2016). The iron silicate was synthesized in the lab following a reported procedure (DeAngelis et al., 2012). Before the fusing process, the Fe_2_SiO_4_ and quartz particles were mixed and ball milled for 12 h. After cooling to room temperature, the resultant product was crushed and sieved to 40–80 mesh.

### Material Characterization

The morphologies of the SCZO and Fe/SiO_2_ samples were visualized using scanning electron microscopy (SEM) on a Hitachi SU-70 electron microscope. N_2_ adsorption–desorption isotherms of both samples were measured using an Autosorb-iQ analyzer (Quantachrome Instruments) at 77 K. The samples were outgassed at 523 K for 8 h and 1 mm Hg prior to measurements. The specific surface areas of the samples were determined using Brunauer–Emmett–Teller (BET) method. The crystalline phases were examined using powder X-ray diffraction (XRD) and obtained on Bruker D8 Advance Lynx Powder Diffractometer (LynxEye PSD detector, sealed tube, Cu Kα radiation with Ni β-filter). The Raman spectra of the coked samples after DNMC reactions with different H_2_ co-feed for time on stream (ToS) of 2 h were collected with a Raman spectrometer (LabRAM Aramis, Horiba Scientific) in the range of 200–2,000 cm^−1^. XPS (ESCALAB 250 Microprobe, Thermo Fisher Scientific) was performed to measure the bonding environment of elements in the fresh and spent SCZO and Fe/SiO_2_ samples.

H_2_-temperature programmed desorption (H_2_-TPD) was performed to determine the H_2_ adsorption on the used perovskite oxide and methane activation catalyst samples. The H_2_-TPD was evaluated using an AutosorbiQ unit (Quantachrome, ASIQM0000-4) equipped with a thermal conductivity detector (TCD). Typically, 100 mg of catalyst sample was loaded into a quartz reactor and pretreated at 973 K for 2 h under He flow (40 ml min^−1^, ultrapure, Airgas) at a heating rate of 10 K min^−1^ from ambient temperature. The sample was then exposed to H_2_ stream (5% H_2_ in nitrogen, 40 ml min^−1^, ultrapure, Airgas) for 0.5 h after being cooled to 363 K under He stream. The physiosorbed H_2_ was removed by flowing He gas (40 ml min^−1^) for 2 h. Next, the sample was ramped to 1223 K at a ramp rate of 10 K min, and the H_2_-TPD profile was recorded during this step.

The temperature-programmed oxidation (TPO) of the spent SCZO and Fe/SiO_2_ samples were acquired using a mass spectrometer (MS, Ametek Proline). In the TPO experiment, 70 mg of spent sample was loaded in a U-shaped tubular quartz reactor (10 mm inner diameter) in which the reactor was placed inside a temperature-controlled furnace (National Electric Furnace FA120 type). The temperature of the furnace was controlled by a Watlow Controller (96 series). A K-type thermocouple was attached to the outer wall of the reactor to monitor the temperature of the catalyst environment. The temperature was increased linearly from room temperature to 1173 K at a ramp rate of 10 K min^−1^ and was held constant for 30 min at the final temperature. A mixture of O_2_/He (30 ml min^−1^; 2% O_2_ and 98% He; Airgas) was introduced by He and sent via heated transfer lines hold at 343 K to the reactor during ramping process. The carbon monoxide (CO) and carbon dioxide (CO_2_) effluents as a function of temperature were analyzed using the mass spectrometer to obtain the TPO profiles.

### Catalytic Direct Non-Oxidative Methane Conversion Reactions

The DNMC catalytic reaction was performed using the same reactor setup as that for TPO, except that the effluents were analyzed using a gas chromatograph (Agilent Technologies, 6890N) equipped with a ShinCarbon ST packed column connected to a TCD and a DB-WAX column connected to a flame ionization detector (FID). The DNMC reactions were carried out at 1273 K and 1 atm pressure. The performance of DNMC on the mixture of SCZO perovskite oxide and Fe/SiO_2_ catalyst were measured by arranging 0.375 g of Fe/SiO_2_ and 0.1875 g of SCZO powder samples in three different ways in the fixed-bed reactor: 1) SCZO oxide on top of the Fe/SiO_2_ catalyst, 2) SCZO oxide at the bottom of the Fe/SiO_2_ catalyst, and 3) well mixing of SCZO oxide and Fe/SiO_2_ catalyst. CH_4_ (18 ml min^−1^, 99.999% purity, Airgas) diluted in N_2_ (as internal standard) (2 ml min^−1^, 99.95% purity, Airgas) was fed to the reactor via heated transfer lines to avoid aromatics condensation. The effects of H_2_ addition on DNMC over the SCZO, Fe/SiO_2_, and their mixture were also investigated by introducing varied H_2_ concentrations to the methane feed stream.

## Results and Discussion

### Physicochemical Properties of SCZO Perovskite and Fe/SiO_2_ Catalyst

**FIGURE 1:**
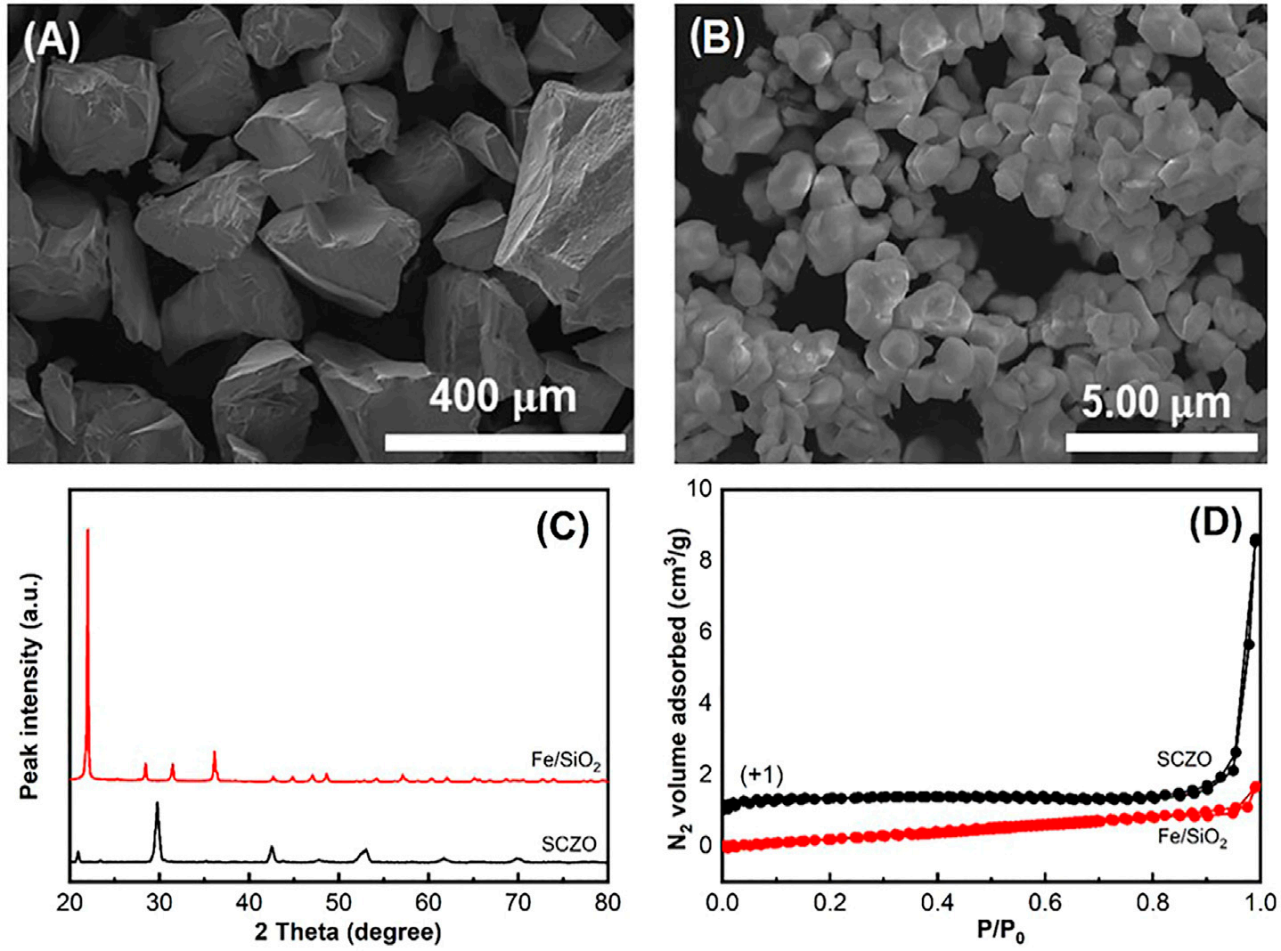
SEM images showing morphologies of Fe/SiO_2_ catalyst **(A)** and SCZO perovskite oxide **(B)** particles. **(C)** and **(D)** are the XRD data and N_2_ adsorption–desorption isotherms of both materials.

The morphologies of both SCZO perovskite oxide and Fe/SiO_2_ catalyst materials were examined by SEM observations. Figures 1A, B show that both materials contain irregular-shaped particles, while the particle size of Fe/SiO_2_ catalyst is ∼300 times larger than the SCZO material. The average particle sizes of Fe/SiO_2_ and SCZO particles are ∼300 μ m and ∼1 μ m, respectively. The crystalline phases of both materials were indicated by XRD data in Figure 1C. The diffraction peaks of Fe/SiO_2_ sample are characteristic of cristobalite phase. No XRD peaks related to iron species was identified. The SCZO oxide has the orthorhombic perovskite structure. No secondary phase in this perovskite material was observed. Figure 1D shows the surface area of both materials. The BET surface areas of Fe/SiO_2_ catalyst and SCZO oxide are 0.38 and 1.01 m^2^ g^−1^, respectively. Both materials have low surface areas.

**FIGURE 2:**
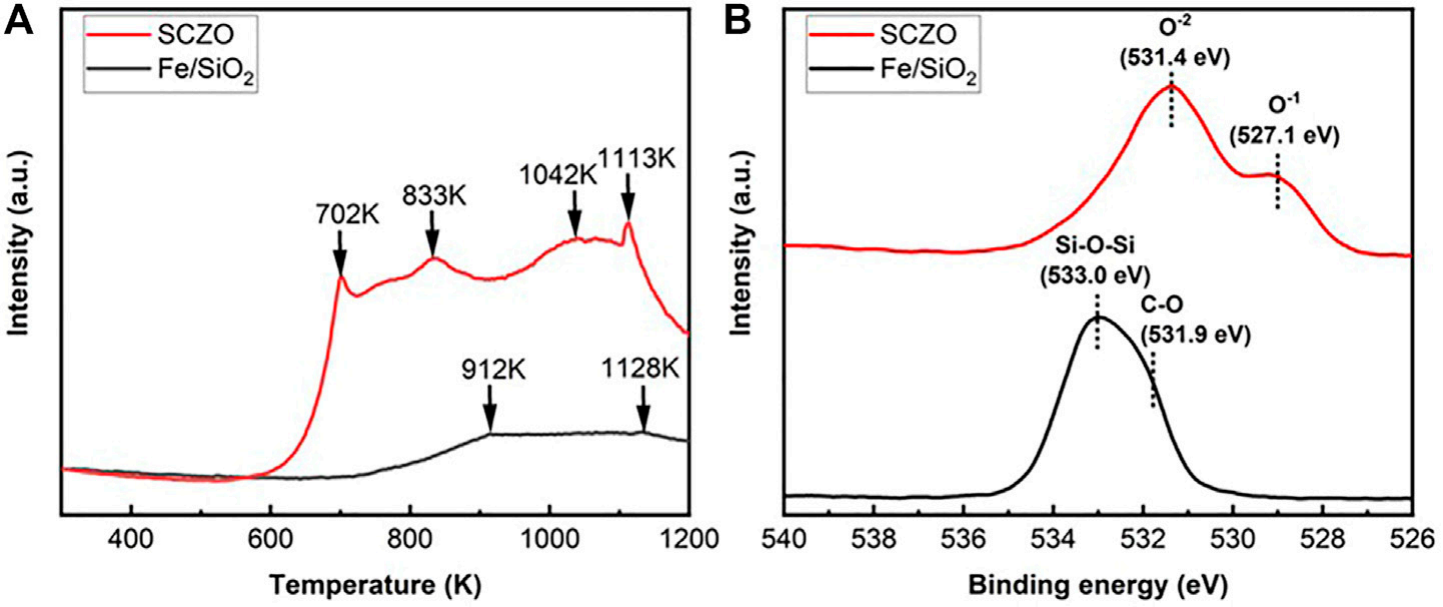
H_2_-TPD profiles **(A)** and XPS spectra of O1s photoelectron lines **(B)** of Fe/SiO_2_ catalyst and SCZO perovskite oxide materials.

The hydrogen adsorption and oxygen coordination environment in both SCZO perovskite and Fe/SiO_2_ catalyst materials were characterized by H_2_-TPD and XPS measurements. As shown in Figure 2A, the H_2_-TPD peaks of SCZO oxide sample are more pronounced than those of Fe/SiO_2_ catalyst. The four peaks at ∼702, ∼833, ∼1042, and ∼1113 K stand for different H_2_ desorption states at a broad range of temperatures in the MIEC ceramic. In contrast, only high-temperature H_2_-desoprtion peaks (i.e., 912 and 1128 K) appear in the Fe/SiO_2_ catalyst. The XPS data in Figure 2B show that the fresh Fe/SiO_2_ catalyst exhibits an O1s XPS peak centered at 533.0 eV that can be assigned to the Si-O-Si structure in the quartz support (Zakaznova-Herzog et al., 2005; Tang et al., 2014). The shoulder peak at 531.9 eV can be assigned to the oxygen bonded in the organic C-O structures (Miller et al., 2002). In the SCZO perovskite, two obvious O1s peaks at 531.4 and 527.1 eV were observed, which can be caused by the O^2−^ and O^1−^ ions in the SCZO perovskite oxide, respectively (Dupin et al., 2000; Wu et al., 2015). The lower binding energies of oxygen species in the O1s XPS of SCZO oxide material than that of Fe/SiO_2_ indicates that the SCZO perovskite can be easily reduced compared to the Fe/SiO_2_ catalyst in the DNMC reaction conditions.

### Direct Non-Oxidative Methane Conversion in the Absence of H_2_ Co-feed in Methane Stream

For DNMC in the membrane reactor that is made of H_2_-permeable SCZO oxide membrane tube packed with Fe/SiO_2_ catalyst in the presence of H_2_ sweep gas, a slight increase in methane conversion, tuning product selectivity towards lighter hydrocarbons, and absence of catalyst deactivation were observed, as reported in our previous work (Sakbodin et al., 2016). The purpose of this study is to reveal the mechanism of this unique property of the H_2_-permeable membrane reactor for DNMC. In order to mimic the fact of co-existence of SCZO, Fe/SiO_2_, and H_2_ co-feed factors in the membrane reactor operation conditions, we used the fixed-bed reactor settings in this work to study the performance of DNMC over Fe/SiO_2_, SCZO oxide, the combination of both, and then integration of these three factors in sequence.

### Direct Non-Oxidative Methane Conversion Over Fe/SiO_2_ Catalyst or SCZO Oxide

**FIGURE 3:**
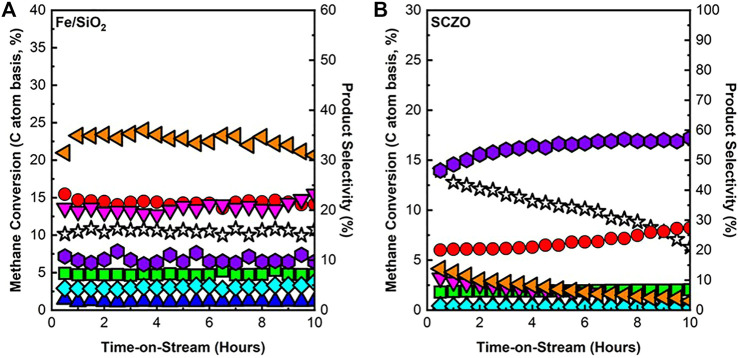
CH_4_ conversion and product selectivity of DNMC over Fe/SiO_2_ catalyst **(A)** and SCZO perovskite oxide **(B)** materials (1273 K temperature, 101.325 kPa pressure, 3,200 mlg^−1^h^−1^ space velocity, molar ratio of N_2_/CH_4_ = 1/9, N_2_ used as internal standard) (symbol indicators: CH4 conversion, acetylene, ethylene, ethane, benzene, toluene, naphthalene, and coke).

The performance of Fe/SiO_2_ or SCZO material in the DNMC reaction with pure methane feed stream was firstly studied. Figure 3 shows the methane conversion and product selectivity versus the ToS of 10 h in both materials. In Figure 3A, DNMC over Fe/SiO_2_ catalyst showed stable methane conversion with no obvious deactivation during the test, in which methane conversion remained at ∼10% with C_2+_ selectivity >90%. There was ∼10% coke formed, but the reaction was independent of coke formation and maintained stable performance, similar to our previous study (Sakbodin et al., 2016). The SCZO material, however, exhibited deactivation over the course of 10-h ToS, as shown in Figure 3B. Methane conversion was high initially (∼13%) but slowly decreased to ∼5% after 10 h of reaction. Coke formation, on the other hand, increased over time, while aromatics products decreased. Up to ∼60% Coke selectivity was observed at ToS of 10 h in DNMC over the SCZO oxide.

The DNMC at the studied conditions involved a complex heterogeneous–homogeneous reaction network (Guo et al., 2014; Toraman et al., 2021). The mechanistic investigation into the Fe/SiO_2_ catalyst revealed that the silica lattice-confined Fe sites initiated CH_4_ dehydrogenation to generate methyl and hydrogen species, enabling a series of subsequent surface and gas-phase reactions to form dehydrogenated and cyclized large hydrocarbon products. The performance of DNMC over the Fe/SiO_2_ catalyst in this study was consistent with our previous work. Although coke was formed in the reaction, the steady state performance data in Figure 3A suggested that a homogeneous gas phase reaction might play a dominant role after DNMC initiation by the heterogeneous catalyst surface. In the SCZO oxide, the high methane conversion at the initial stage of the reaction (e.g., ToS < 2 h) suggested its effectiveness in initiating the methane activation. The high coke selectivity, however, hinted that the catalyst was not effective in suppressing the dehydrogenation of the hydrocarbon intermediates or products, thus causing coke formation. The carbonaceous deposits were detrimental to the SCZO because of the blocked active sites on the SCZO and eventually led to catalyst deactivation, as shown in Figure 3B.

### Direct Non-Oxidative Methane Conversion Over a Mixture of Fe/SiO_2_ Catalyst and SCZO Oxide

**FIGURE 4:**
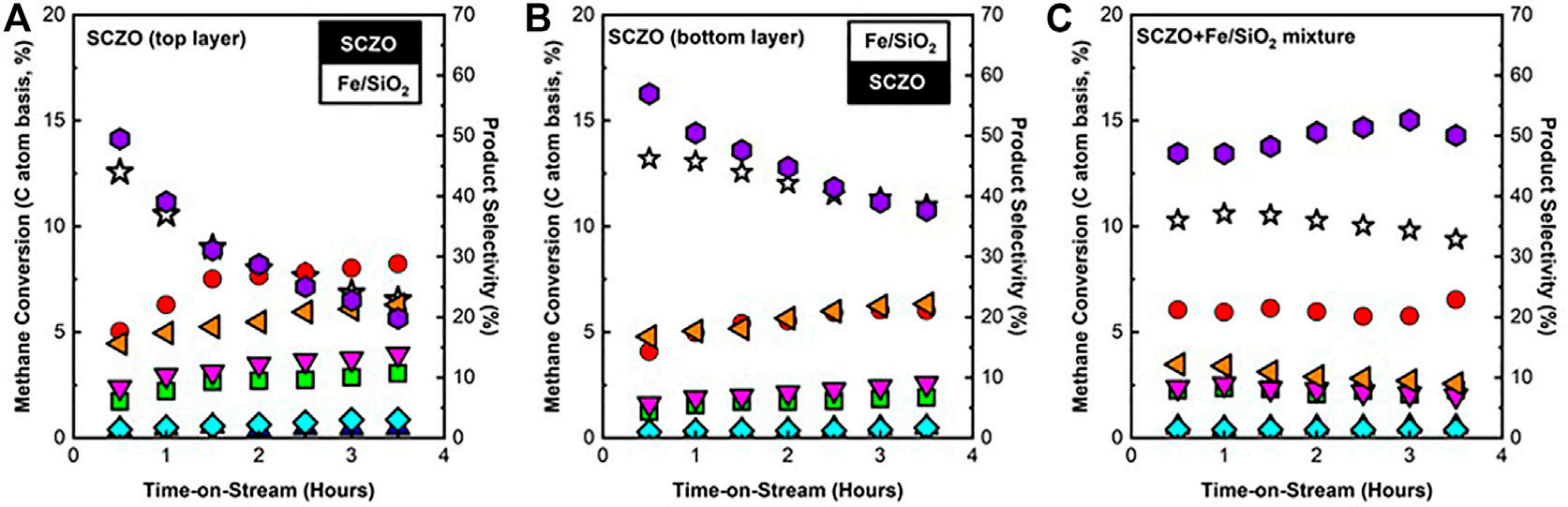
CH_4_ conversion and product selectivity in DNMC reaction over Fe/SiO_2_ catalyst and SCZO perovskite oxide mixture with different sample arrangement format in the reactor: **(A)** SCZO packed on top of Fe/SiO_2_, **(B)** SCZO packed below Fe/SiO_2_, and **(C)** SCZO and Fe/SiO_2_ well mixed (mass ratio of SCZO to Fe/SiO_2_ = 1:2, 1273 K temperature, 101.325 kPa pressure, 2,133 mlg^−1^h^−1^ space velocity, molar ratio of N_2_/CH_4_ = 1/9, N_2_ used as internal standard) (symbol indicators: CH4 conversion, acetylene, ethylene, ethane, benzene, toluene, naphthalene, and coke).

To understand the DNMC performance in the membrane reactor that had SCZO membrane tube packed with Fe/SiO_2_ catalyst internally, the SCZO oxide and Fe/SiO_2_ catalyst samples were arranged in three different manners in the fixed-bed reactor to mimic H_2_-permeable membrane reactor setup for DNMC. As described in an earlier section, the layer of SCZO oxide was placed on the top or bottom or evenly distributed in the Fe/SiO_2_ catalyst layer, respectively. Figure 4A shows the methane conversion and product selectivity when SCZO oxide was placed on top and at the bottom of the Fe/SiO_2_ catalyst, as well as when both SCZO and Fe/SiO_2_ powder were mixed, for ToS of 3.5 h. The overall methane conversion was slightly lower when SCZO was placed on top of the Fe/SiO_2_ layer compared to when SCZO was placed at the bottom of the Fe/SiO_2_ layer. Methane conversion was lower in the first case because methane reacted with the SCZO material first before reaching Fe/SiO_2_ catalyst. As discussed above, the SCZO material promoted coke formation more easily than Fe/SiO_2_ powder. Therefore, coke formed on the SCZO material tended to block the active sites on the Fe/SiO_2_ catalyst located at the bottom layer and reduced methane activation from both layers. In terms of product selectivity, ethylene and acetylene selectivity were higher when SCZO was arranged at the top layer. Since methane reacted on SCZO material through surface reaction, the dehydrogenation of methane on the surface formed not only coke but also C_2_ products. Aromatic products were proposed to form in the gas phase homogeneously through a series of cyclization reactions. The aromatic selectivity was lower in the case when SCZO was placed at the top layer because less methane was reacted with Fe/SiO_2_ catalyst to form reaction intermediates for gas phase cyclization reactions.

When SCZO was placed at the bottom of the Fe/SiO_2_ catalyst, the overall methane conversion and coke selectivity were higher. As shown in Figure 4B, Fe/SiO_2_ catalyst did not show significant deactivation over the course of 10-h reaction. Therefore, when methane reacted with the Fe/SiO_2_ catalyst layer first, C_2_ and higher products were formed through both surface and gas-phase reactions. However, when these products (including C_2_ and aromatics) encountered the SCZO layer at the bottom, they underwent surface reactions on the SCZO oxide to form coke even though methane continued to react at the top Fe/SiO_2_ layer. The higher coke selectivity and lower C_2_ and higher hydrocarbon selectivity in such SCZO and Fe/SiO_2_ sample arrangement verify the proposed surface kinetics of SCZO and both surface and gas phase kinetics of Fe/SiO_2_ catalyst. In addition, comparing both catalyst arrangements in Figures 4A, B, methane deactivated faster when the SCZO material was located on the top layer. As explained earlier, methane reacted with SCZO first to form coke, which in turn led to catalyst deactivation. As for the case when both SCZO and Fe/SiO_2_ powder were physically well mixed (Figure 4C), methane conversion showed only very slight deactivation from ∼11% to ∼9.5% over the course of 3.5 h. Coke selectivity also increased at a slower rate compared to the previous two cases. Such catalyst arrangement allowed methane to react with SCZO and Fe/SiO_2_ powder at the same probability. The coke formed through surface reaction on SCZO material again blocked the active sites of the Fe/SiO_2_ catalyst, causing slight deactivation on the overall methane conversion. Overall, a synergetic interaction between these two materials in close proximity has led to more stable DNMC performance compared to the other two arrangement modes of SCZO and Fe/SiO_2_ materials.

### Direct Non-Oxidative Methane Conversion in the Presence of H_2_-Cofeed in Methane Stream

#### Direct Non-Oxidative Methane Conversion Over Fe/SiO_2_ Catalyst or SCZO Oxide With H_2_ Co-feed

**FIGURE 5:**
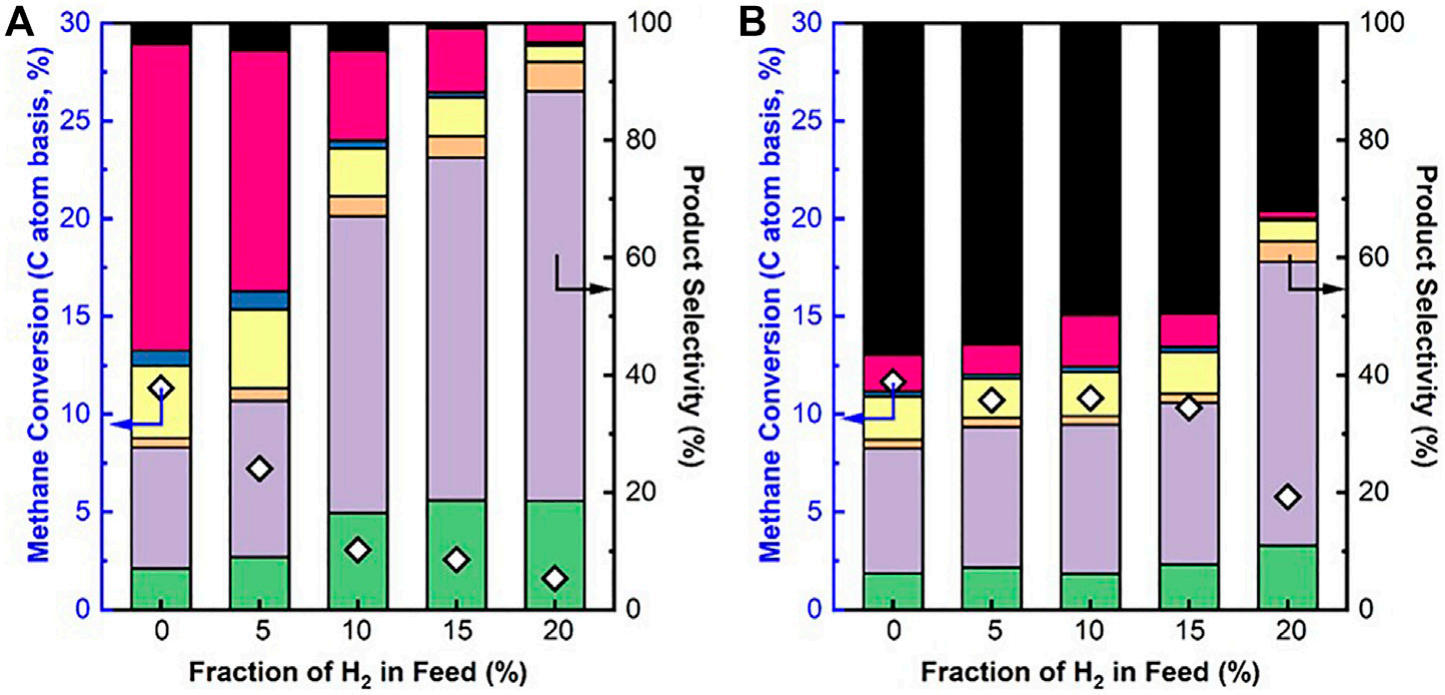
CH_4_ conversion and product selectivity in DNMC over Fe/SiO_2_ catalyst **(A)** and SCZO perovskite oxide **(B)** in a fixed-bed reactor at different hydrogen co-feed concentrations (1273 K temperature, 101.325 kPa pressure, 3,200 mlg^−1^h^−1^ space velocity, molar ratio of N_2_/CH_4_ = 1/9, N_2_ used as internal standard, ToS of 1 h) (symbol indicators: coke, naphthalene, toluene, benzene, ethane, ethylene, and acetylene.

To understand the effects of H_2_ sweep gas on the DNMC in the membrane reactor, the DNMC over SCZO oxide or Fe/SiO_2_ catalyst in the presence of H_2_ co-feed was studied. Figure 5 presents the methane conversion and product selectivity in DNMC over each of these two materials at different H_2_ co-feed concentrations at ToS of 1 h. Methane conversion over the Fe/SiO_2_ catalyst decreased with increasing H_2_ co-feed concentration (Figure 5A) due to the reverse reaction of DNMC, consistent with Le Chatelier's principle. The product selectivity shifted from heavy aromatics to light hydrocarbons. The coke formation was also decreased. These results are consistent with previous reports on DNMC over the metal/zeolite catalysts (Olsvik and Billaud, 1993; Olsvik and Billaud, 1994). It should be noted that the degree of methane conversion was dropped significantly with the H_2_ co-feed concentration. For example, at 10% of the H_2_ co-feed, methane conversion was dropped to ∼2.5%, about four times lower than that in the absence of H_2_ co-feed.

The presence of H_2_ co-feed, however, did not show significant detrimental impact on methane conversion in DNMC over the SCZO oxide, except that the H_2_ co-feed was a very high (e.g., 20%) condition. Figure 5B shows that methane conversion was kept at ∼12% when no H_2_ was added to the reaction. When H_2_ co-feed concentration increased from 5% to 15%, methane conversions were maintained at ∼11%. Unlike Fe/SiO_2_ catalyst, coke selectivity remained almost the same at around 52–58% for SCZO material, except for 20% H_2_ co-feed concentration where the selectivity dropped more significantly. The C_2_ product selectivity, on the other hand, increased slightly with increasing H_2_ co-feed concentration. The DNMC performance data over these two materials verified that SCZO oxides favored surface reaction by cleaving the C-H bond in methane to form C_2_ products and coke, instead of both surface and gas-phase reaction in the case of DNMC on the Fe/SiO_2_ catalyst. The H_2_ co-feed in the reaction system likely did not influence the surface reaction significantly, while it eliminated the gas-phase reaction rapidly during the DNMC in the studied conditions.

#### Direct Non-Oxidative Methane Conversion Over a Mixture of Fe/SiO_2_ Catalyst and SCZO Oxide With H_2_ Co-feed

**FIGURE 6:**
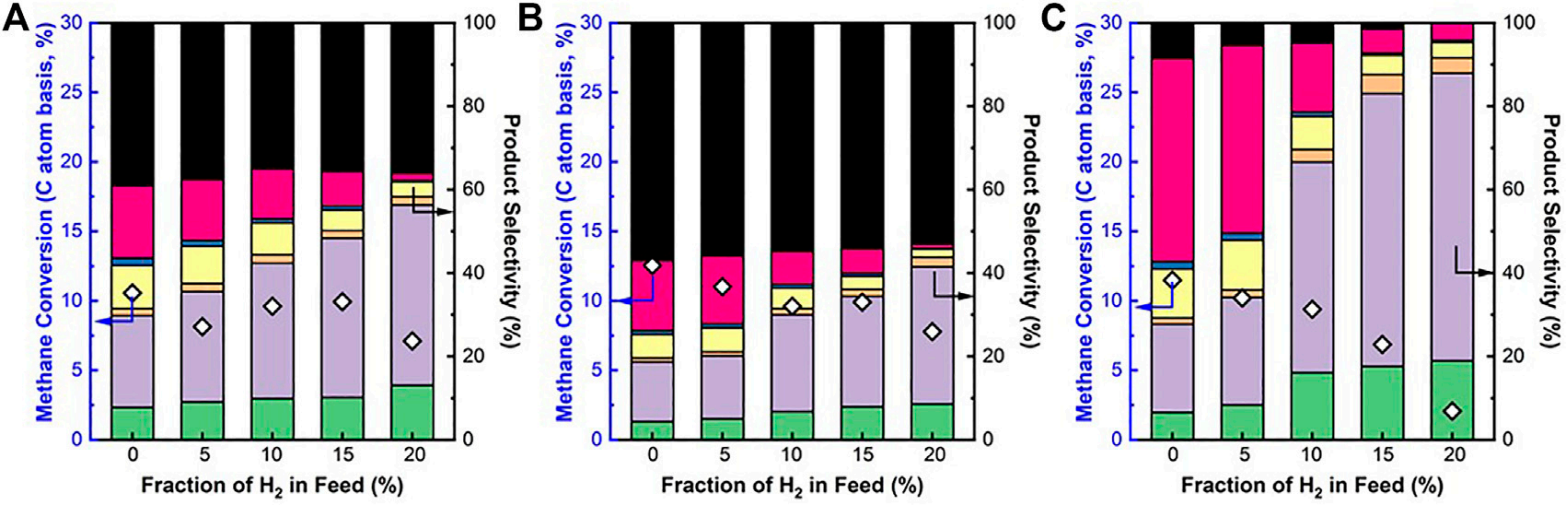
CH_4_ conversion and product selectivity in DNMC reaction over Fe/SiO_2_ catalyst and SCZO perovskite oxide mixture with different sample arrangement: **(A)** SCZO packed on top of Fe/SiO_2_, **(B)** SCZO packed below Fe/SiO_2_, and **(C)** SCZO and Fe/SiO_2_ well mixed [mass ratio of SCZO to Fe/SiO_2_ = 1:2 in **(A)** and **(B)** and 5:95 in **(C)**, 1273 K temperature, 101.325 kPa pressure, 2,133 mlg^−1^h^−1^ **(A)** and **(B)** and 3,200 mlg^−1^h^−1^ **(C)** space velocities, molar ratio of N_2_/CH_4_ = 1/9, N_2_ used as internal standard, ToS of 1 h] (symbol indicators: coke, naphthalene, toluene, benzene, ethane, ethylene, and acetylene).

The effects of H_2_ co-feed on the catalytic activity and product selectivity of the Fe/SiO_2_ catalyst and SCZO oxide materials at three different sample mixing modes were studied. When SCZO oxide stayed on the top of the Fe/SiO_2_ catalyst, the methane conversion was ∼10%, independent of H_2_ concentration until 20% H_2_ co-feed in the methane stream was used (Figure 6A). This result is very similar to the case that only SCZO oxide was used in the reactor as shown in Figure 5B. The coke selectivity decreased slightly with increasing hydrogen concentration. Overall, the coke selectivity was lower compared to the testing condition that only SCZO oxide was used in the reactor. Figure 6B shows the methane conversion and product selectivity of DNMC when SCZO oxide was placed below the Fe/SiO_2_ catalyst. Similarly, the addition of H_2_ in the methane feed decreased methane conversion and increased light C_2_ product selectivity. The coke selectivity was high compared to that in Figure 6A, which should be caused by the severe secondary and the following on reactions of the products that are formed from the top Fe/SiO_2_ catalyst layer. Overall, the presence of SCZO oxide in the reactor maintained stable methane conversion except for the conditions with high H_2_ co-feed. At the same time, the usage of SCZO oxide led to high coke selectivity.

Figure 6C showed the DNMC performance in the well-mixed SCZO oxide and Fe/SiO_2_ catalyst samples in the reactor. It should be noted that the amount of SCZO oxide in the Fe/SiO_2_ catalyst bed was only 5wt%, about 6.6 times lower than the SCZO amount in the first two mixing modes. The reduction in SCZO oxide usage is based on two considerations. Firstly, SCZO seems to be very active in methane activation that leads to coke formation easily. The lower usage of SCZO is expected to lower the coke formation. Secondly, the 5wt% SCZO oxide has a comparable surface area of SCZO membrane that had contacted with Fe/SiO_2_ catalyst in the membrane reactor settings in our previous work (Sakbodin et al., 2016). The surface area ratio between SCZO oxide and Fe/SiO_2_ catalyst in this test was controlled to be the same as that in the membrane reactor condition. In contrast to the previous two SCZO oxide and Fe/SiO_2_ catalyst mixing modes, methane conversion dropped gradually from ∼11% to ∼3% when H_2_ co-feed concentration increased from 0% to 20%. Coke selectivity also decreased, while heavy aromatics selectivity shifted to lighter hydrocarbons when more H_2_ was added to the reaction. However, a closer look showed that methane conversion did not decrease as sharply as the case that only Fe/SiO_2_ catalyst was used in Figure 5A. In particular, when H_2_ co-feed concentrations were 5% and 10%, high methane conversions and low coke selectivity were reached compared to the sole presence of Fe/SiO_2_ catalyst or SCZO oxide. The H_2_ co-feed sharply reduced methane conversion on the Fe/SiO_2_ catalyst (Figure 5A), but it mildly decreased methane conversion on the well-mixed SCZO oxide and Fe/SiO_2_ catalyst mixture (Figure 6C). As the MIEC conductor, it is expected that SCZO oxide functions as a “hydrogen transformer” that absorbs co-fed hydrogen and produce activated hydrogen species to reduce coke formation in the DNMC on the Fe/SiO_2_ catalyst. This function could lower the local hydrogen species concentration that is relevant to the DNMC reaction in the reactor and thus lessens the reverse reaction of DNMC according to the La Chatelier's principle.

### Characterization of Spent Fe/SiO_2_ Catalyst and SCZO Oxide Materials

#### Raman Spectroscopy

**FIGURE 7:**
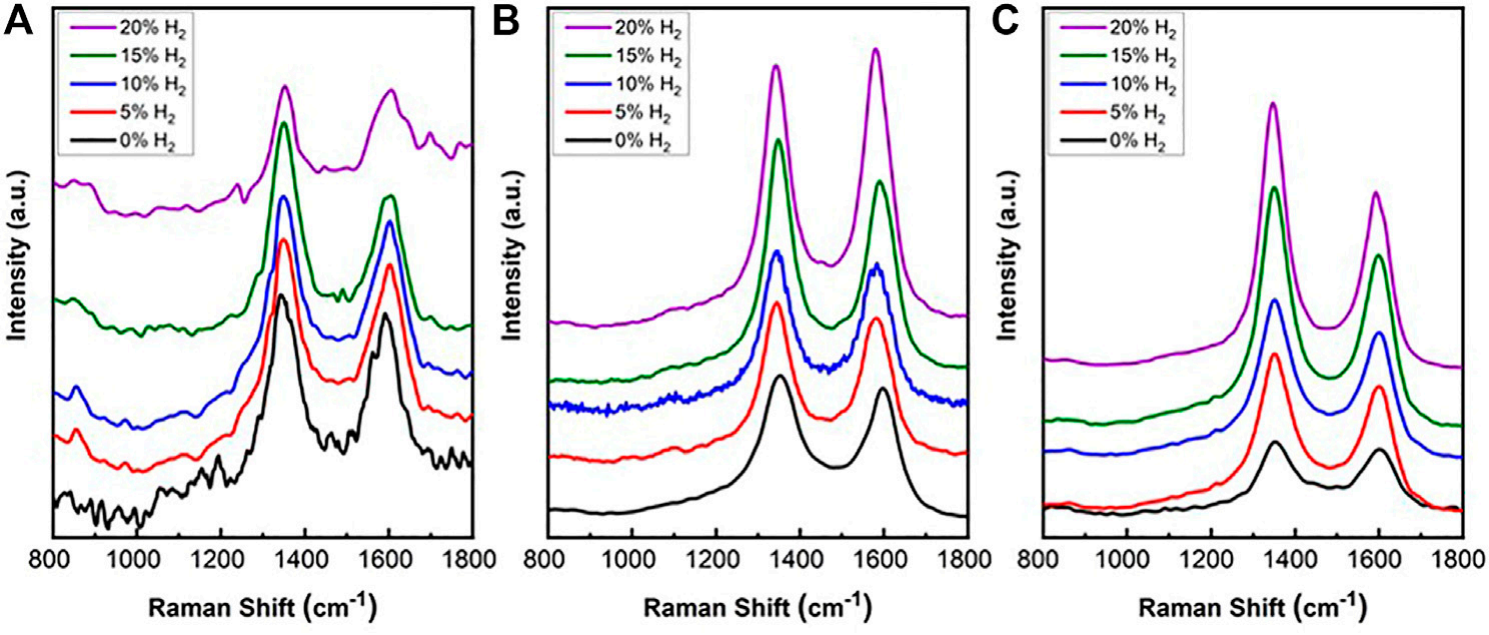
Raman spectra of coke formed on spent Fe/SiO_2_ catalyst **(A)**, SCZO perovskite oxide **(B)**, and Fe/SiO_2_ catalyst mixed with 5 wt% SCZO perovskite **(C)**, respectively, in DNMC reaction in a fixed-bed reactor at different hydrogen co-feed concentrations after ToS of 3.5 h.

Raman spectra were measured from spent SCZO, Fe/SiO_2_, and their mixture (i.e., 5wt% SCZO in Fe/SiO_2_) materials after ToS of 3.5 h in DNMC at different H_2_ co-feed concentrations, and the results are shown in Figure 7. Nearly no fluorescence background was detected in all these spectra. The Raman analysis confirmed the existence of two types of carbon structures. The spectra of all the spent catalysts are similar with two observed peaks centered at 1,320 and 1,600 cm^−1^, respectively. The band at 1,320 cm^−1^ is assigned to D band, while the band at 1,600 cm^−1^ is assigned to G band (Espinat et al., 1985; Bare et al., 2017). D band represents disordered graphitic structure, amorphous carbon, or polyaromatic type species, while G band relates to graphite involving out-of-phase intra-layer displacement in the graphene structure (Roeges, 1994). There is no significant shift in the two peaks as a function of SCZO oxide usage and H_2_ co-feed concentration.

Table 1 shows the ratio of D band to G band of coke formed on all the samples at different H_2_ co-feed concentration. In the absence of H_2_ co-feed, the ratios are similar among Fe/SiO_2_ catalyst, SCZO perovskite oxide, and their mixture. After the addition of H_2_ co-feed, the ratio increased in the Fe/SiO_2_ sample, decreased in the SCZO sample, and increased more obviously in the Fe/SiO_2_ and SCZO mixture sample, although the ratio did not show obvious dependence on the H_2_ co-feed concentrations in each case. These results suggested that more coke of ordered graphitic structure was formed on the pure SCZO oxide sample, while more coke of amorphous types was formed in the pure Fe/SiO_2_ catalyst. The mixing of both materials in the reactor, however, facilitated the formation of amorphous types of coke slightly.

**TABLE 1:** Ratio of D band to G band determined from Raman spectroscopy analysis for Fe/SiO_2_ catalyst, SCZO perovskite oxide, and 5 wt% SCZO oxide mixed with 95 wt% Fe/SiO_2_ catalyst after 3.5 h DNMC reaction at 1273 K and at different H_2_ co-feed concentrations.

H_2_ co-feed (%)	D to G band ratio		
	Fe/SiO_2_	SCZO	5 wt% SCZO in Fe/SiO_2_
0	1.25	1.30	1.24
5	1.33	1.11	1.37
10	1.39	1.14	1.45
15	1.58	1.21	1.46
20	1.34	0.92	1.59

### Temperature Programmed Oxidation

**FIGURE 8:**
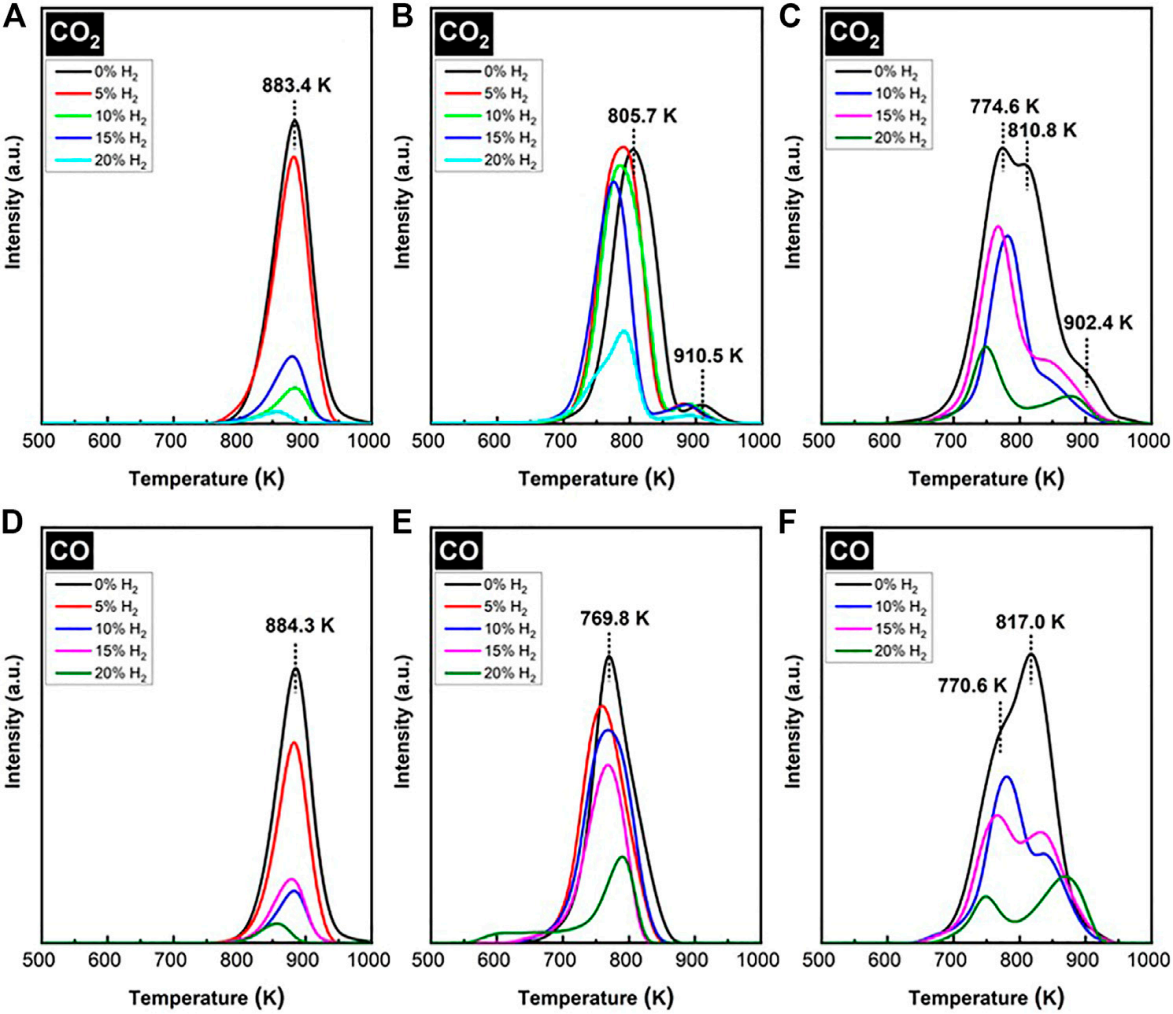
CO_2_ and CO evolution peaks in TPO profiles of spent Fe/SiO_2_ catalyst **(A** and **D)**, SCZO perovskite oxide **(B** and **E)**, and 5wt% SCZO in Fe/SiO_2_ **(C** and **F)** at different H_2_ co-feed concentrations after ToS of 3.5 h in DNMC reactions.

The TPO of spent SCZO oxide, Fe/SiO_2_ catalyst, and their mixture in the DNMC reactions were measured, and the results are shown in Figure 8. The MS signals observed in the TPO profiles include CO_2_ and CO; no water signal was detected. Therefore, it was assumed that the coking species on the spent catalysts should be primarily carbonaceous. The CO_2_ and CO from the combustion of spent Fe/SiO_2_ catalyst displayed relatively narrow TPO profiles (Figures 8A, D), with an onset of 750 K and completion occurring at 1000 K, a span of 250 K. The H_2_ co-feed decreased the peak intensity and shifted the peak maximum to lower temperatures. On average, the peak maximum stayed around 860 K. At 20% H_2_ co-feed concentration, the sharp decrease in CO_2_ peaks was consistent with the sharp decrease in methane conversion in DNMC shown in Figure 5A. For the CO_2_ and CO profiles from spent SCZO oxide (Figures 8B, E), both TPO peaks spanning from 670—950 K were broadened compared to those of spent Fe/SiO_2_ catalyst, and the peak maximum shifted to 800 K in the CO_2_ and 760 K in CO profiles. In the CO_2_ effluent profiles, a small shoulder peak (centered at 900 K) appeared in the high-temperature region. The peak intensity did not reduce obviously with H_2_ co-feed until the 20% H_2_ concentration was used in the DNMC reaction. These results showed that SCZO oxide was more active in methane activation, and it was not influenced by the additional H_2_ presence due to its MIEC property. For coking species on the spent SCZO oxide and Fe/SiO_2_ mixture sample, the TPO profiles (Figures 8C, F) seem to be the sum of profiles of the two individual materials. The peak spanned broadly from 650 to 1000 K, and the peak maximum stayed at 770 K with very clear shoulder peaks at the high-temperature end. The H_2_ co-feed decreased the peak intensity but not as strongly as the cases in the Fe/SiO_2_ sample.

The TPO peak temperatures reflected the types of coking species in the spent methane activation catalysts. The TPO spectra for each spent catalyst could be deconvoluted into three peaks: a low-, medium-, and high-temperature peak. The low-temperature peak could be described as amorphous and oxidized “soft” coke, the medium-temperature peak to polymeric aromatic carbon, and the high-temperature peak to the ordered, graphitic “hard” coke. The formation of diverse carbon species in the DNMC reaction has been observed in Mo/ZSM-5 (Liu H. et al., 2002; Ma et al., 2002; Song et al., 2014) and metal/sulfated zirconia catalysts (Abedin et al., 2019; Abedin et al., 2019; Kanitkar et al., 2019) as well as in methane pyrolysis in the absence of any catalyst (Gueret et al., 1995; Vander Wal et al., 2018; Singh et al., 2019; Wang et al., 2019). The H_2_ co-fed DNMC condition in the presence of SCZO oxide apparently leads to an obvious increase in the soft coke on the Fe/SiO_2_ catalyst, resulting in active DNMC reaction without obvious methane conversion drop or high coke selectivity.

### X-Ray Photoelectron Spectroscopy

**FIGURE 9:**
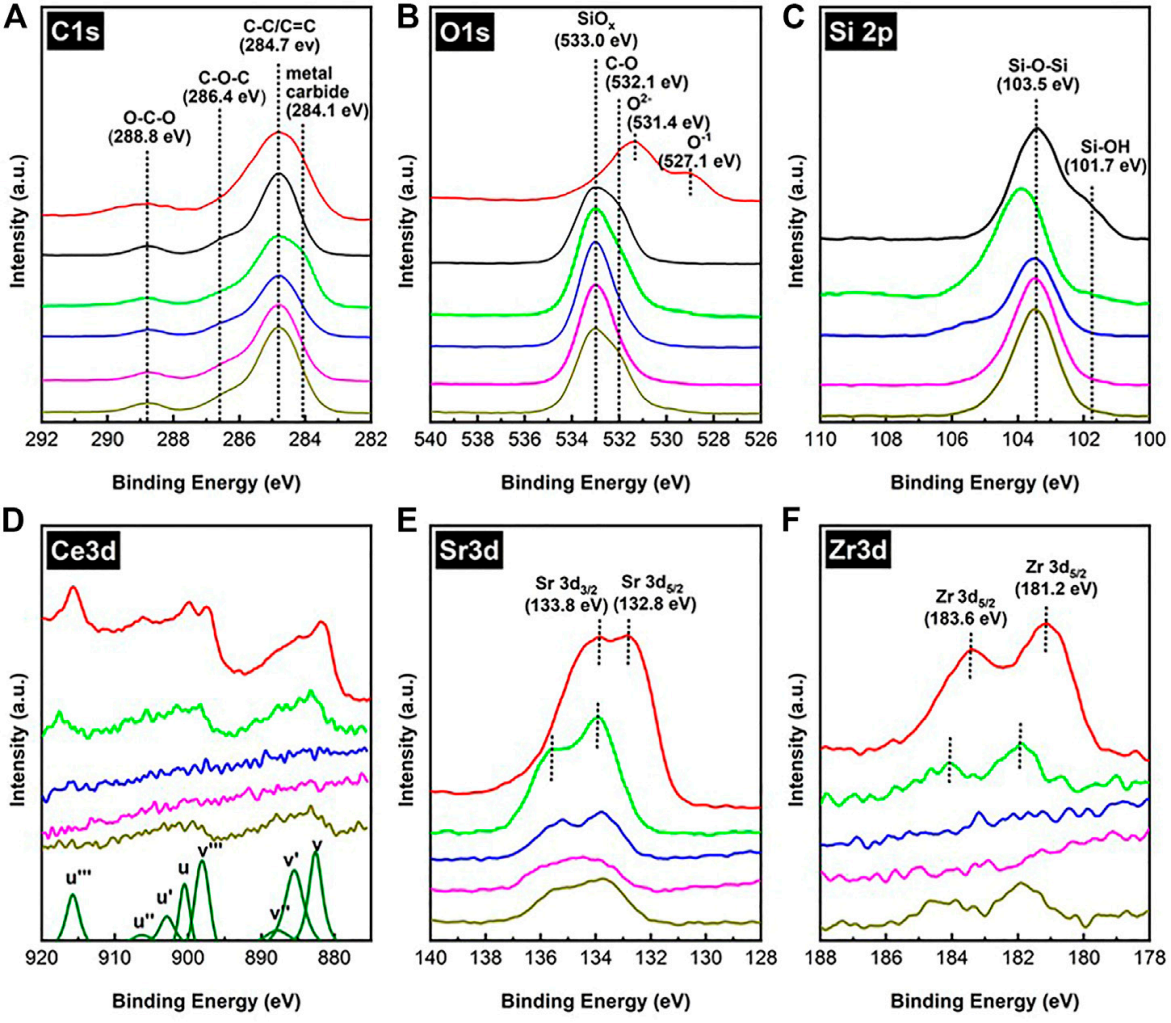
XPS spectra of C1s **(A)**, O1s **(B)**, Si2p **(C)**, Ce3d **(D)**, Sr3d **(E)**, and Zr3d **(F)** of fresh SCZO (red), fresh FeSiO_2 (_black), spent FeSiO_2_ mixed with SCZO in 2:1 mass ratio (green), spent FeSiO_2_ mixed with 5 wt% SCZO (blue), spent FeSiO_2_ mixed with 5 wt% SCZO under 10% H_2_ co-feed (purple), and spent FeSiO_2_ mixed with 5 wt% SCZO under 20% H_2_ co-feed (dark yellow), respectively.

Figure 9 shows the XPS spectra of C 1s, O 1s, Si 2p, Ce 3d, Sr 3d, and Zr 3d of the spent Fe/SiO_2_ and SCZO mixture samples in DNMC in the absence and presence of H_2_ co-feed conditions. The XPS spectra of fresh SCZO or Fe/SiO_2_ were included in some of the sub-figures in Figure 9 for comparison purpose. In Figure 9A, the XPS data of C 1s from the fresh SCZO and Fe/SiO_2_ samples has prominent peaks, which are attributed to adventitious carbon present on the surface of the as-prepared materials (Barr and Seal, 1995; Budde et al., 2018). The XPS spectra of C1s can be deconvoluted into four peaks, located at 284.1, 284.7, 286.4, and 288.8 eV in sequence, which can be assigned to carbon in the carbidic (e.g. Si-C or Fe-C), graphitic (C-C or C=C), C-O-C, and O-C-O structures, respectively (Miller et al., 2002). In comparison to individual SCZO oxide or Fe/SiO_2_ catalyst sample, after the DNMC reaction, the XPS peak assigned to C-C or C=C group in the SCZO and Fe/SiO_2_ mixture samples increased significantly, suggesting the carbon deposition onto these samples from DNMC. The appearance of peak around 284.1 eV suggested the formation of metal carbide species when SCZO amount was high in the mixture. In 5wt% SCZO in Fe/SiO_2_ sample, this low binding energy peak diminished, so the XPS spectra of C 1s from the SCZO and Fe/SiO_2_ mixture sample shares the same feature as that of the fresh SCZO and Fe/SiO_2_, regardless the H_2_ concentration in the feed stream.

The XPS data of O 1s in Figure 9B shows that the fresh Fe/SiO_2_ exhibits two peaks at 533.0 and 532.1 eV, which could be assigned to the Si-O-Si structure in the quartz support (Zakaznova-Herzog et al., 2005; Tang et al., 2014) and the oxygen bonded in the organic C-O structures (Miller et al., 2002), respectively. The two peaks at 531.4 and 527.1 eV in fresh SCZO were assigned to the O^2−^ and O^1−^ ions in the perovskite oxide material, as discussed in the *Physicochemical Properties of SCZO Perovskite and Fe/SiO*_*2*_ *Catalyst* section. After the DNMC reaction, in the SCZO and FeO_2_ mixture sample, the XPS peaks of C-O and oxide ions were reduced significantly. The reductive environment apparently removed these oxygen species. In the presence of 20% H_2_ co-feed, this XPS peak recovers its intensity to the level similar to that of the fresh Fe/SiO_2_ sample. Figure 9C presents the XPS data of Si 2p in theses samples. The peaks at 103.5 and 101.7 eV in Fe/SiO_2_ sample are associated to the Si-O-Si and Si-OH structures. The positions of peak at 130.5 eV are shifted to higher binding energies because of the Si-O-Si bond interaction with the metal-oxygen bonds in SCZO in their mixture (33wt% SCZO in Fe/SiO_2_) in DNMC (Dane et al., 2006). Different from shifting Si 2p to higher binding energies in the mixture with high SCZO usage, 5wt% SCZO in Fe/SiO_2_ did not have obvious peak shift. The presence of H_2_ in the methane feed, however, reduced the Si-OH structure, but Si-O-Si was not influenced obviously.

As a reducible metal oxide, the XPS data of Ce 3d, Sr 3d, and Zr 3d in SCZO perovskite oxide were analyzed to understand their changes caused by the DNMC reaction. As shown in Figure 9D, the Ce 3d spectrum of the fresh SCZO sample has complicated features due to mixing of Ce 4f levels with O 2p states. Two sets of spin-orbital multiplets, corresponding to the 3d_3/2_ and 3d_5/2_ contributions, were labeled as u and v, respectively (Burroughs et al., 1976; Silvestre-Albero et al., 2002; Reddy et al., 2003). The peaks labeled v and v′′ have been assigned to a mixing of Ce 3d^9^ 4f^2^ O 2p^4^ and Ce 3d^9^ 4f^1^ O 2p^5^ Ce^4+^ final states, and the peak denoted v′′′ corresponds to the Ce 3d^9^ 4f^0^ O 2p^6^ Ce^4+^ final state. On the other hand, the peak v′ is assigned to Ce 3d^9^ 4f^1^ O 2p^6^ of Ce^3+^. The same assignment can be applied to the u structures, which correspond to the Ce 3d_3/2_ levels. The very sharp v, v′′′, u, and u′′′ peaks indicated the Ce^4+^ dominantly existing in the fresh SCZO. In the spent SCZO and Fe/SiO_2_ mixture samples, all these peaks have reduced intensity and shifted to higher binding energies. The same phenomenon was observed for Sr 3d and Zr 3d XPS data in Figures 9E, F. The decrease in the peak intensity was caused by the low SCZO concentrations in the mixture samples, while the peak shift should be due to the bonding environment change.

In general, a pseudo-binary metal oxide alloy formed by mixing two elemental oxides (e.g., M_a_O_b_ and N_m_O_n_) displays M-O-M, N-O-N, and M-O-N bonds. If the electronegativity of atom N was larger than M, there were M–O alloy bonds that were more ionic and N–O bonds that were more covalent in the alloys than in the respective elemental oxide phases (Rayner Jr, 2002). In the SCZO and SiO_2_ mixture, Si was more electronegative than any metal atoms in SCZO. Therefore, the Ce-O bond was more positively ionic than its state in the SCZO material alone, and thus the peak shifted to a higher binding energy. The same reasoning applies to the left-shift in binding energies of Sr 3d and Zr 3d in Figures 9E, F. In the spent SCZO and Fe/SiO_2_ mixture (i.e., 33wt% SCZO in Fe/SiO_2_), the v′ and u′ peaks are very obvious, indicating the presence of Ce^3+^ in the sample. The decrease in SCZO quantity to 5wt% decreased the peak intensity of Ce 3d XPS peak in the SCZO and Fe/SiO_2_ mixture sample. The peak intensity is too low to detect clear peaks in the spent sample. The presence of 20% H_2_ co-feed in the methane stream in the reaction enabled appearance of these peaks, but the peak intensity is quite low to inform any confirmative information. For the same reason, the XPS peaks of Zr 3d were not obvious for clear analyses. As noted earlier, the formation of Zr-O-Si bond in the SCZO and Fe/SiO_2_ mixture is responsible for shifting the Sr 3d peaks to higher binding energies. The presence of H_2_ co-feed does not show obvious influences on its bonding environment. Due to the low concentration (0.075 wt%) of Fe in the Fe/SiO_2_ catalyst, the peaks correlating to Fe binding energies showed a low signal-to-noise ratio and were not included in this discussion.

## Conclusion

The DNMC was studied in a fixed-bed reactor packed with a well-mixed Fe/SiO_2_ catalyst and SCZO perovskite oxide materials. By flowing proper concentration of H_2_ co-feed in the methane stream, DNMC with stable and high methane conversion and low coke selectivity were achieved. Characterizations on the spent Fe/SiO_2_ catalyst, SCZO oxide, and their mixture samples show the co-existence of Fe/SiO_2_, SCZO, and H_2_ co-feed favors low-temperature “soft coke” formation. As a MIEC material, SCZO could function as a “hydrogen transformer” that converts absorbed hydrogen into smaller species (e.g., proton and electron) to influence coke formation on the catalyst. This leads to a low local H_2_ concentration and less potential to reverse the DNMC reaction according to the Le Chatelier's principle. The addition of H_2_ co-feed into the reactor packed with the sole Fe/SiO_2_ catalyst, however, leads to a sharp decrease in methane conversion. The present study mimics DNMC in the H_2_-permeable membrane reactor that is made of SCZO membrane tube packed with Fe/SiO_2_ catalyst with H_2_ sweep gas flowing outside. The results obtained in this work can guide the design and operation of H_2_-permeable membranes for alkane dehydrogenation in general.

## Data Availability

The original contributions presented in the study are included in the article/Supplementary Material; further inquiries can be directed to the corresponding author.
